# Molecular Research of Regulation of Red Blood Cells in Health, Hereditary or Acquired Diseases

**DOI:** 10.3390/ijms25116174

**Published:** 2024-06-04

**Authors:** Jean-Luc Wautier

**Affiliations:** Faculté de Médecine, Université Denis Diderot Paris, 75013 Paris, France; jltwautier@gmail.com

During the first era of humanity, the conditions of life, including hunting, fighting, obtaining food, and diseases, were associated with frequent hemorrhages, anemia, and infections, which led to death or untreatable conditions. However, blood diseases are now a major problem, which are the result of excessive blood cell production [1].

This Special Issue focuses on blood component production and regulation, as well as some of the functions of various blood components, which have not been thoroughly explored. Since the discovery of microscopes, major progress has been achieved regarding blood cell identification and the understanding of their functions. Genetic analysis and molecular biology deepened our understanding of blood cell functions and the regulation of hematopoiesis. Hematopoiesis is a tightly regulated mechanism of blood cell production and release in the bloodstream. This process originates from hematopoietic stem cells (HSCs) and extent through to lineage-committed progenitors, which produce mature leukocytes, red blood cells, and platelets [2]. The development of specific lineages is regulated through intrinsic factors, including combinations of transcription factors and epigenetic modifications, along with extrinsic cues such as cytokines and growth factors [3].

Hematological abnormalities and diseases were previously classified according to clinical features or blood cell examination. This classification was revised to also consider cell receptor defects, genetic mutations, regulation by cytokines, and other regulating factors.

The consequences of blood cell abnormalities on organ functioning have been extensively studies, opening the way for new active therapies.

Erythrocytes, which play a major role in oxygen transport, are important in blood circulation, thrombosis, and vascular diseases. In the treatment of inherited red blood cell disorders, hemoglobinopathy is largely dependent upon blood transfusion, which is not always an effective method. However, new approaches using stem cell transplantation show promise as treatments for these disorders [4].

## 1. Environment

Environmental conditions such as oxygen pressure can influence hematopoiesis. However, these factors have different effects in hematopoiesis according to certain genetic characteristics, as observed in the Himalayan mountains. Oxygen (O_2_) deprivation creates stress and is paradoxically linked to the accumulation of free radicals. The adaptative response during hypoxia starts with a temporary arrest during the cell cycle, leading to reduced energy consumption and the secretion of proangiogenic factors. The response involves various cellular pathways, including the gene regulation of hypoxia-inducible factors (HIFs). HIFs exist in three isoforms: HIF1α, HIF2α, and HIF3α. Additionally, the activities of the prolyl hydroxylase domain-containing enzyme PHD3 are regulated by O_2_ availability [5].

## 2. Genetic Markers

In hematopoietic stem cells (HCSs), somatic mutations can accumulate with each division, with most divisions having no effect; however, some result in the initiation of programmed cell death [6]. Other HSC mutations evade clearance and can result in competitive advantages, which are characterized by increased self-renewal, proliferation, survival, and biased lineages. HSCs likely acquire one to two mutations per division, which equates to approximately 10 mutations/year when extrapolated. Modeling results suggest that most of our HSCs have two coding mutations once we reach adulthood, suggesting that each HSC is unique and keeps acquiring mutations at this life stage. These mutations can result in clonal hematopoiesis of indeterminate potential (CHIP). Individuals with CHIP are at an increased risk of mortality, which is now linked to cardiovascular disease. CHIP mutations may be associated with thrombotic disease; the mechanisms contributing to this pathology and potential therapies require further study. Somatic mutations that can result in several hematological disorders. CHIP occurs when HSCs acquire a somatic mutation that provides them with a competitive growth advantage over normal HSCs. This results in a relatively higher number of mutated hematopoietic cells in the bone marrow and blood. The number of white blood cells is only slightly dependent on somatic mutations. The proportion of mutated cells in the blood in CHIP is affected by age. One possible explanation for this is the insufficient repair of damaged DNA. Less than 5% of individuals under 60 years of age display CHIP, whereas approximately 10% is observed in subjects over 60 years old. This prevalence continues to increase with age. Sequencing results have indicated that hematological somatic leukemogenic mutations indicative of CHIP are most commonly observed in the genes *DNMT3A*, *TET2*, *ASXL1*, *JAK2*, and *TP53*. Mutations in the epigenetic modifier DNMT3A can lead to CHIP [2].

Myeloproliferative neoplasms (MPNs), also called BCR-ABL-MPN, are characterized by the excessive production of differentiated blood cells, which leads to polycythemia vera, (PV) essential thrombocythemia (ET), and primary myelofibrosis. PV is the most common MPN. The JAK2V617F mutation occurs in a pluripotent progenitor, a hematopoietic progenitor existing in almost all myeloid lineages. However, this mutation is absent in nonhematopoietic cells but found in the endothelial cells of the spleen and splanchnic vessels. Discovery of the genetic mutations responsible for hematological neoplasms simplified the strategy for diagnosing myeloproliferative syndromes and leukemia [7].

A deeper understanding of the functions of adhesion molecules either in genetic or acquired disorders has opened a new possibility: blocking antibodies or peptides can be used to prevent thrombosis or limit cancer cell extension.

The relatively easy access to the markers present in the blood has enabled more timely cancer detection. Chemical and biological components offer the opportunity to more selectively limit blood cell production using enzyme blockers or monoclonal antibodies. Hematopoietic growth factors can be used to limit the side effects of chem therapies and reduce the need for blood transfusion [8].

Erythropoietin (EPO) was initially proposed as a treatment for anemia secondary to renal failure. EPO is also administered to reduce the need for transfusion in surgery and to extend the possibility of autotransfusion. Patients in the preoperative setting and, more frequently in the postoperative period, suffer from anemia (10% to 90% of cases). In patients undergoing surgery, blood cell counts and blood transfusion tests are systematically prescribed. When anemia is found, the cause should be explored, and cardiac tolerance should be evaluated. Several strategies can be applied to reduce the need for blood transfusion, including preoperative treatment with vitamins and iron, limiting blood loss during surgery, hemodilution, and autotransfusion.

The progress achieved in the genetic field or the molecular defect at the origin of the blood disease it has major consequences on the diagnostic strategy and treatment.
